# Nematicidal Effects and Cytotoxicity of Levamisole on *Thelazia callipaeda*

**DOI:** 10.3390/ani15111551

**Published:** 2025-05-26

**Authors:** Zhengxuan Han, Yipeng Zhong, Ni Chen, Zichen Liu, Zhankui Yuan, Yipeng Jin

**Affiliations:** 1 College of Veterinary Medicine, China Agricultural University, Yuanmingyuan West Road 2, Haidian District, Beijing 100193, China; h401201814@163.com (Z.H.); fuyingsiren@yeah.net (N.C.); lzc94@126.com (Z.L.); 2 College of Veterinary Medicine, Nanjing Agricultural University, Chinese Nanjing Weigang No. 1, Nanjing 210095, China; 9211710424@stu.njau.edu.cn

**Keywords:** *Thelazia callipaeda*, *Thelazia callipaeda* viability assessment system, levamisole, topical ocular deworming, experimental animal infection model, in vivo efficacy, serum biochemical analysis, complete blood count (CBC), ocular symptom scoring, RCEC, cytotoxicity

## Abstract

*Thelazia callipaeda* (*T. callipaeda*) is a zoonotic ocular parasite that poses a public health risk. Current treatment depends on mechanical parasite extraction and specific prophylactic anthelmintics, but direct ocular deworming agents are limited and expensive, and mechanical removal is often incomplete, causing animal stress and ocular injury. Levamisole eye drops have been applied; however, their efficacy and safety remain undefined. We established a standardized *T. callipaeda* viability assessment system (TVAS) and an animal infection model to evaluate candidate drugs. In vivo, 5 mg/mL levamisole was administered at escalating frequencies. Ocular symptoms, complete blood count (CBC), and serum biochemistry were measured, and cytotoxicity was assessed using Cell Counting Kit-8 (CCK-8) and lactate dehydrogenase (LDH) assays on rabbit conjunctival epithelial cells (RCECs).Four doses of 5 mg/mL levamisole applied at 30 min intervals cleared *T. callipaeda* from infected eyes within 2 h without significant changes in CBC, serum biochemistry, or ocular symptom scores. CCK-8 and LDH assays indicated minimal cytotoxicity in RCECs within 4 h. However, prolonged exposure (6–12 h) led to a significant decrease in RCEC viability, suggesting potential cytotoxicity with extended use and highlighting the need for further safety evaluation. A regimen of four topical administrations of 5 mg/mL levamisole at 30 min intervals cleared *T. callipaeda* from infected eyes with minimal cytotoxicity, supporting its safety and efficacy as a topical treatment.

## Simple Summary

*Thelazia callipaeda* infection is a significant concern. We developed a *Thelazia callipaeda* viability assessment system and an experimental animal model to facilitate its research. There are limited ophthalmic deworming treatments for *Thelazia callipaeda* infection. This study explored an effective administration method and the in vivo and in vitro safety of levamisole, a deworming drug traditionally used by Chinese veterinarians.

## 1. Introduction

*Thelazia callipaeda* (*T. callipaeda*), a nematode belonging to the order *Spirurida*, family *Thelaziidae*, and genus *Thelazia*, is an ocular parasite that utilizes *Phortica* spp. as intermediate hosts. *T. callipaeda* can infect humans and various mammals, parasitizing the conjunctival sac and causing thelaziasis [1,2]. Due to the prevalence of thelaziasis in East Asian countries such as China, Japan, and South Korea, *T. callipaeda* is also referred to as the oriental eyeworm [3,4]. Cases of *T. callipaeda* infection in humans, domestic animals, and wildlife have been reported in Asian countries such as China, Japan, South Korea, and India, as well as in several European countries and the northeastern United States, with prevalence continuing to rise, which poses a zoonotic disease of public health risk [1,3,5,6,7,8,9,10,11].

Veterinarians routinely use sterile cotton swabs and saline irrigation to extract parasites from the conjunctival sac, supplementing this with anthelmintic treatment for prevention and therapy. Recent research has focused on therapeutic agents for *T. callipaeda*, notably an eye drop formulation of ivermectin (10 mg/mL, 6 μg per dose) diluted in 10% propylene glycol, which has demonstrated effective deworming [12]. Milbemycin and moxidectin are currently approved in Europe for managing canine ocular *T. callipaeda* infection [13,14]. Sarolaner/moxidectin/pyrantel (Simparica Trio^®^), given orally at monthly intervals, is also effective in preventing *T. callipaeda* infection [15]. One study evaluated the efficacy of a combination of esafoxolaner, eprinomectin, and praziquantel (NexGardÒ Combo) for treating *T. callipaeda* in naturally infected cats [16]. However, ocular treatment options via eye drops are limited; a single dose of 2.5% moxidectin combined with 10% imidacloprid was found to achieve 100% elimination of ocular parasites in infected animals within 7 days [17]. However, dedicated ocular drop formulations are currently unavailable in some Asian countries. Chinese veterinarians have employed levamisole solution as an ophthalmic treatment for this disease. As early as the 1990s, 0.5% levamisole hydrochloride eye drops were used to treat affected dogs, while 0.25% levamisole hydrochloride was used for cats [18]. A 0.5–1% levamisole ophthalmic solution is employed to treat *T. callipaeda* infection in cattle and sheep [19]. By approximately the 2010s, affected dogs received a 7-day continuous ocular treatment with 5% levamisole hydrochloride eye drops [20,21,22].

After establishing the *T. callipaeda* viability assessment system (TVAS), this study investigated the most effective in vivo dosing regimen of levamisole against conjunctival *T. callipaeda*, confirmed the ocular and systemic signs induced by topical eye-drop administration, and evaluated its effects on the proliferation and metabolic activity of conjunctival epithelial cells.

## 2. Materials and Methods

### 2.1. Material

The live *T. callipaeda* samples used in this study were collected from canines diagnosed with thelaziasis at the China Agricultural University Veterinary Teaching Hospital. The canines were all naturally infected, with cases recorded from May 2021 to July 2024.

The RCECs used in the experiments were purchased from Shanghai Baiye Biotechnology Center, Shanghai, China.

### 2.2. Establishment of the T. callipaeda Viability Assessment System (TVAS)

In this study, we established the *T. callipaeda* viability assessment system (TVAS) by adapting widely used manual motility assays in *Caenorhabditis elegans*—specifically, counting C-shaped thrashes in liquid medium over 30 s (thrashing assay) [23], recording dorsal–ventral body bends on solid agar within 30 s (body-bend assay) [24], and manually scoring static morphological features such as curvature and rigidity [25]. TVAS evaluates worm viability by the total movement count and morphological assessment over a 30 s interval. The detailed scoring criteria are presented in Table 1(1-1), with point values in Table 1(1-2).

### 2.3. Investigation of the Nematicidal Effect of Levamisole on T. callipaeda

#### 2.3.1. Development of Animal Models for *T. callipaeda* Maintenance and Infection

Rabbits served as maintenance hosts, with clinical *T. callipaeda* specimens inoculated into their conjunctival sacs for parasite maintenance. Twelve clean-grade New Zealand White rabbits (2.5 ± 0.3 kg, 3–4 months old) were selected as experimental animals. Routine ophthalmic evaluations—including visual inspection, Schirmer tear test (without anesthesia), and fluorescein staining with 15 s exposure—confirmed their ocular health, and the animals were then randomized into four groups (*n* = 3 per group). Six viability-scored (score = 8) *T. callipaeda* worms were collected from maintenance rabbits and inoculated into one eye of each test rabbit—care was taken to prevent worms from escaping via the medial canthus. Twenty-four hours post-inoculation, successful parasitism was confirmed by macroscopic observation and worm counting. No severe adverse reactions occurred, and all rabbits were included in the analysis.

#### 2.3.2. In Vivo Levamisole Anthelmintic Experiment Against *T. callipaeda*

Levamisole hydrochloride (CAS no. 16595-80-5) was dissolved in DMEM to prepare a 0.5% solution. The solution was freshly prepared before each use, protected from light, and stored at 4 °C until application.

There were four experimental groups that received 0.5% levamisole solution, with treatments applied 1, 2, 3, or 4 times, and a control group receiving no treatment. Each administration consisted of 50 µL, with rabbits passively closing their eyes for 10 s. Treatments were applied at 30 min intervals, with three *T. callipaeda*-infected rabbit models per group. Worm detachment was monitored at 1 h, 2 h, 4 h, 8 h, and 12 h post-treatment and compared to baseline. Worm viability was scored according to Table 1, and ocular symptoms were recorded based on Table 2. CBC and serum biochemical analysis were conducted on 6 rabbits pre- and post-experiment.

### 2.4. Cytotoxicity Assessment of Levamisole on RCECs

#### 2.4.1. Standard Culture of RCECs

RCECs obtained commercially were observed under an inverted microscope to ensure >90% confluence. The old medium was replaced, cells washed twice with PBS, digested with trypsin, and resuspended in complete DMEM to prepare a cell suspension for passage. Cells were incubated at 37 °C with 5% CO_2_, and passages 10–30 were selected for experiments.

#### 2.4.2. CCK-8 Assay

RCEC cell suspension was prepared and seeded into 96-well culture plates for 24 h. Cells were divided into experimental, control, and blank control groups. The experimental group was treated with 100 µL of a levamisole hydrochloride–DMEM medium pre-mixture containing 0.5% levamisole. The control group was treated with 100 µL of DMEM medium, and the blank group contained only DMEM medium without cells. Each group had 6 replicates. After 2 h of incubation, 10 µL of CCK-8 reagent was added to each well and incubated for another 2 h in a CO_2_ incubator. The optical density (OD) of each well was measured at 450 nm, with a microplate reader being used to calculate the cell proliferation rate.

The cell proliferation rate was measured after 2 h, 4 h, 6 h, and 12 h of levamisole treatment using the same method. The procedure in each group was performed in triplicate.

#### 2.4.3. LDH Cytotoxicity Assay

Samples were prepared and grouped as described in Section 2.4.2. After 2 h of incubation in a cell culture incubator, the 96-well plate was centrifuged at 5 min to remove the supernatant. Each well was then filled with 150 µL of PBS-diluted cell lysis buffer (1:10 dilution) and incubated for 1 h. After centrifugation for 5 min, 120 µL of supernatant was transferred to a new 96-well plate. Subsequently, 60 μL of LDH detection solution was added to each well, and the plate was incubated for 30 min in a cell culture incubator. The OD was measured at 490 nm with a microplate reader to determine intracellular LDH activity. Intracellular LDH activity was measured at 2 h, 4 h, 6 h, and 12 h after levamisole treatment following the same procedure. Each experimental group was tested in triplicate.

### 2.5. Statistical Method

All statistical analyses and graphical representations were conducted using GraphPad Prism version 9.5 (GraphPad Software, San Diego, CA, USA). Data are expressed as the mean ± standard deviation (SD). The normality of data distribution was assessed using the Shapiro–Wilk test, and the homogeneity of variances was evaluated using Levene’s test. For comparisons involving more than two groups, one-way analysis of variance (ANOVA) was performed. When data met the assumptions of normality and equal variances, ANOVA was followed by Dunnett’s post hoc test to compare each treatment group with the control group, thereby controlling the family-wise error rate. For comparisons between two groups, an unpaired two-tailed Student’s *t*-test was used when variances were equal; otherwise, Welch’s *t*-test was applied.

A *p*-value < 0.05 was considered statistically significant, and *p* < 0.01 was considered highly significant.

## 3. Results

### 3.1. In Vivo Lethal Effect of Levamisole on T. callipaeda

**Figure 1:**
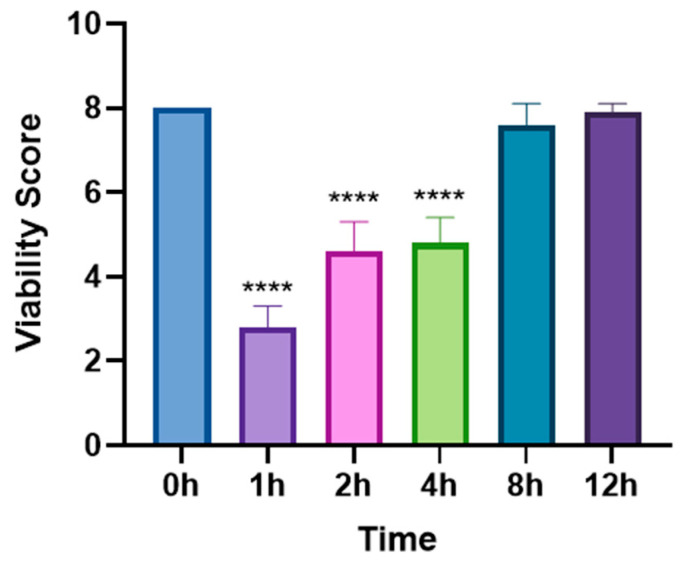
Motility scores of *Thelazia callipaeda* at different time points after a single levamisole administration. Each experimental rabbit had one eye infected with six *Thelazia callipaeda* (motility score = 8), and the experiment was repeated in three groups. Levamisole eye drops (5 mg/mL) were applied once. The motility scores of retained nematodes were assessed at different time points and compared with the scoring data at 0 h. **** *p* < 0.0001.

**Figure 2:**
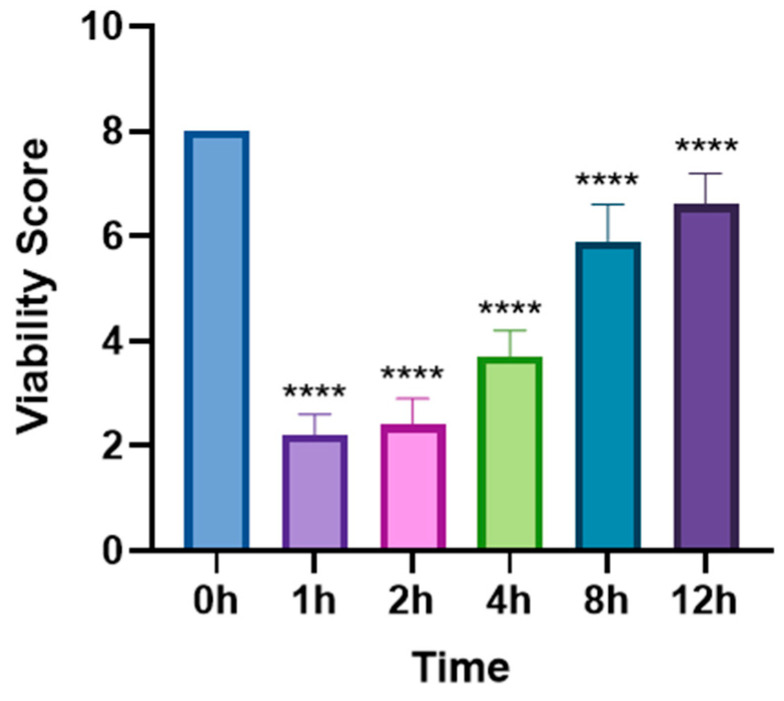
Motility scores of *Thelazia callipaeda* at different time points following two administrations of levamisole eye drops. Levamisole eye drops (5 mg/mL) were applied twice at a 30 min interval. **** *p* < 0.0001.

**Figure 3:**
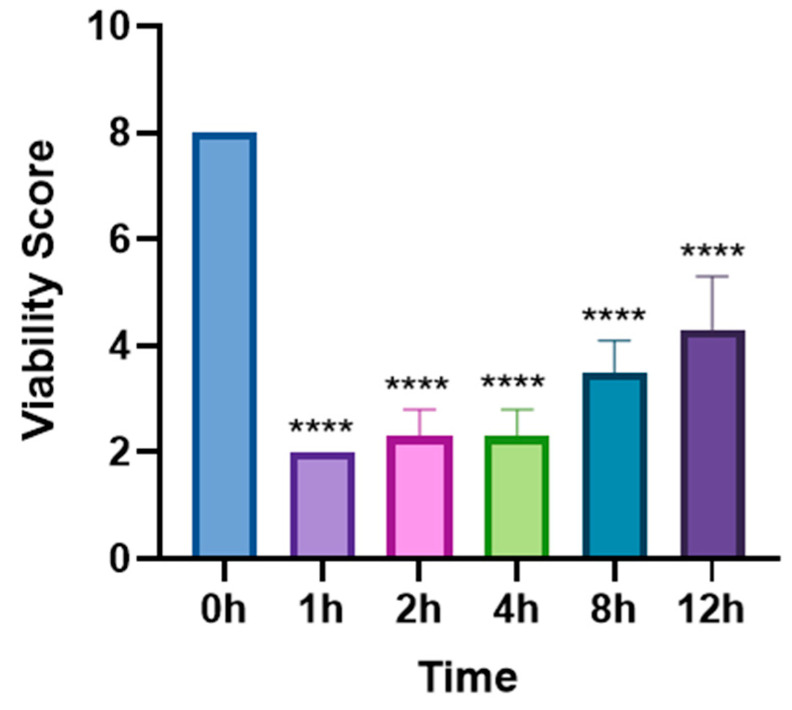
Motility scores of *Thelazia callipaeda* following three doses of levamisole at different time points. Levamisole eye drops (5 mg/mL) were applied three times at a 30 min interval. **** *p* < 0.0001.

**Figure 4:**
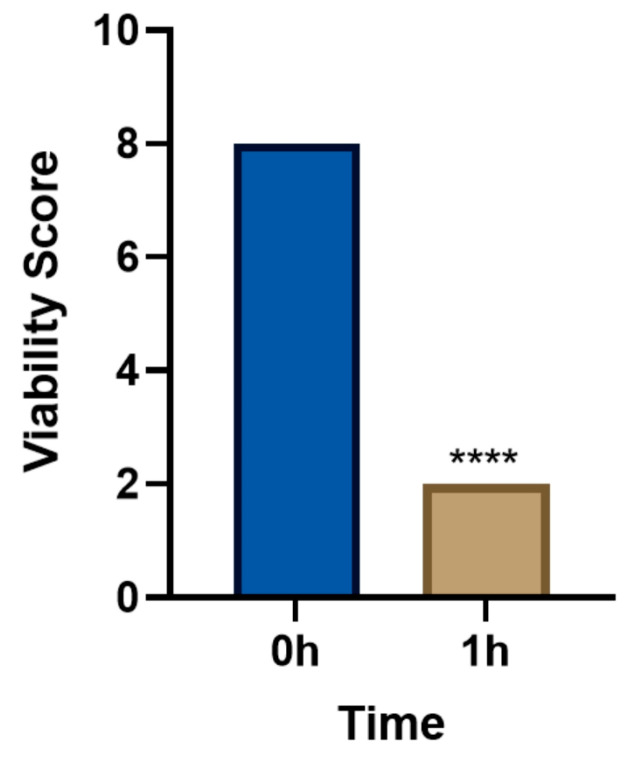
Motility scores of *Thelazia callipaeda* following four administrations of levamisole at different time points. Levamisole eye drops (5 mg/mL) were applied four times at a 30 min interval. **** *p* < 0.0001.

**Figure 5:**
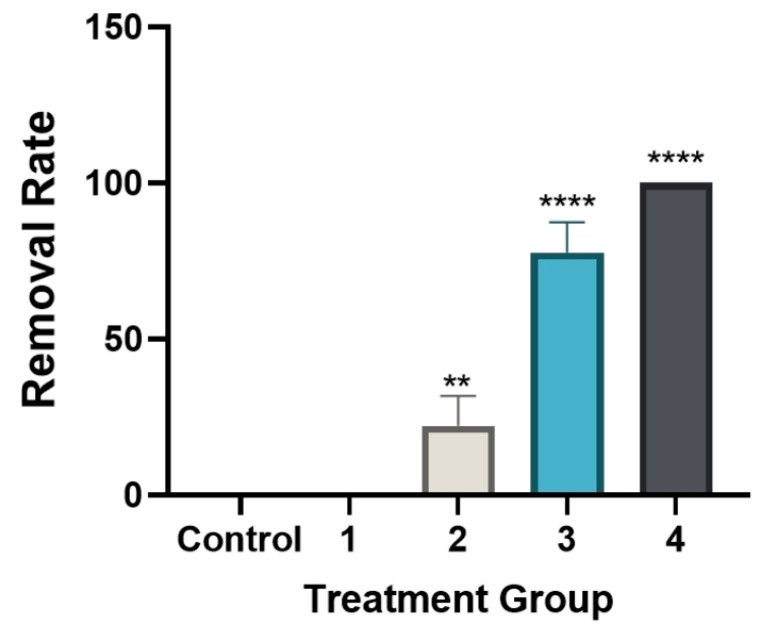
Effect of 5 mg/mL levamisole on nematode detachment rate with different dosing frequencies. Nematode detachment rate was calculated as detached nematodes/total infected nematodes (%) (*n* = 3 per group). The control group and the single-dose group showed 0% detachment, while increasing dosing frequency led to a dose-dependent increase in detachment. ** *p* < 0.01; **** *p* < 0.0001.

**Figure 6:**
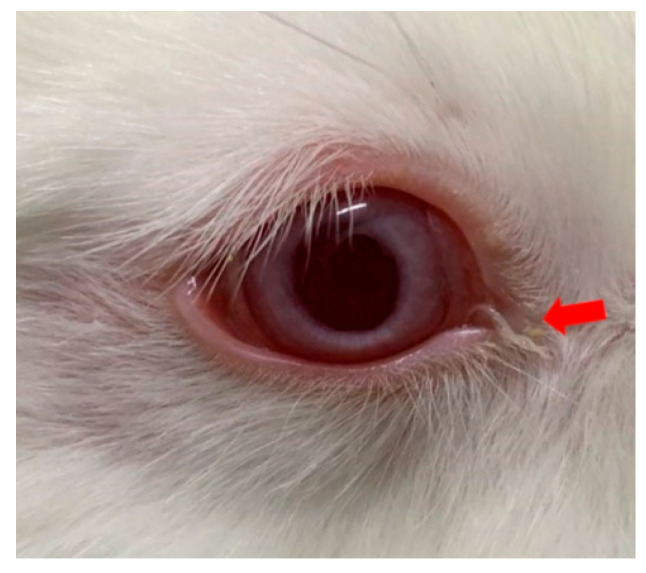
Expelled *Thelazia callipaeda* in the process of detachment. Following *Thelazia callipaeda* death or reduced viability, blinking of the experimental rabbit pushes them toward the medial canthus. The arrow marks the nematodes in the process of detachment.

In the single-dose group (5 mg/mL levamisole), the motility score of *T. callipaeda* significantly decreased at 1 h, 2 h, and 4 h post-administration but recovered by 8 h (Figure 1). However, no worms were expelled. In the two-dose group, the motility score remained significantly reduced within 12 h post-administration (Figure 2), and some worms were expelled in all three trials. In the three-dose group, the motility score dropped even further within 12 h post-administration (Figure 3), and the expulsion rate was higher compared to that of the two-dose group. In the four-dose group, the motility score decreased significantly within 1 h, and the expulsion rate was the highest (Figure 4). All worms were expelled within 2 h. The expulsion rates for all treatment groups are shown in Figure 5, while the detached or immobilized worms expelled by rabbit blinking are shown in Figure 6.

### 3.2. Ocular Symptoms Scoring in Experimental Animals Following Levamisole Administration

Eight hours post-infection with *T. callipaeda*, conjunctival symptom scores in the affected eyes significantly increased and secretion enhanced. In the single-dose treatment group, both conjunctival symptoms and secretion scores showed a significant rise at 8 h post-administration. The two-dose treatment group exhibited a significant increase in conjunctival symptom scores after 8 h, whereas the three-dose treatment group showed a significant increase at 12 h. No abnormalities were observed in the four-dose treatment group. Detailed scoring results are presented in Table 3.

### 3.3. Hematological and Biochemical Assessments in Experimental Animals Following Levamisole Administration

Hematological and biochemical parameters were evaluated in six experimental rabbits following four doses of levamisole. No significant differences were observed between pre-treatment and 24 h post-treatment values (*p* > 0.05), and all values remained within the normal reference range. Data were compared using the independent samples *t*-test. The detailed results are presented in Table 4 and Table 5.

### 3.4. Time-Dependent Changes in Cell Proliferation Following Levamisole Treatment

**Figure 7:**
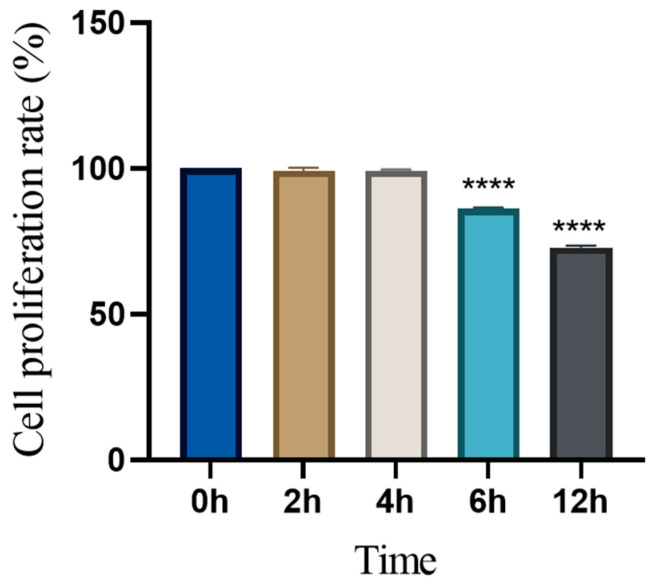
Effect of 5 mg/mL of levamisole on conjunctival epithelial cell viability over time. Cell viability was assessed using the CCK-8 assay at 0 h, 2 h, 4 h, 6 h, and 12 h after treatment with 5 mg/mL of levamisole. The absorbance at 450 nm was measured and normalized to the 0 h control group, which was set as 100%. **** *p* < 0.0001.

The cell viability of RCECs treated with 5 mg/mL of levamisole at different time points (0 h, 2 h, 4 h, 6 h, and 12 h) was evaluated using the CCK-8 assay. As shown in Figure 7, no significant decrease in cell viability was observed up to 4 h of treatment. However, after 6 h of treatment, a significant reduction in cell viability was observed (*p* < 0.05), which continued at 12 h. The cell viability at 12 h was approximately 72.9% that of the control group, with a marked decrease compared to that at earlier time points. These results suggest that the 5 mg/mL levamisole treatment did not induce significant cytotoxicity within the first 4 h but did lead to a slight reduction in cell viability after prolonged exposure.

### 3.5. Changes in Relative LDH Activity (%) Following Levamisole Treatment

**Figure 8:**
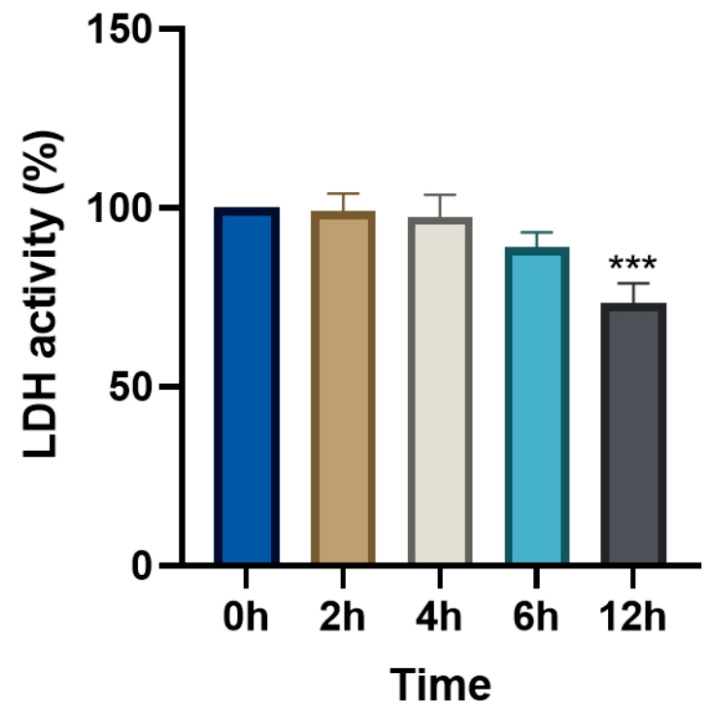
Effect of 5 mg/mL of levamisole on conjunctival epithelial cell cytotoxicity over time. LDH activity in RCECs following treatment with 5 mg/mL of levamisole at various time points. Data are expressed as the relative LDH activity (%) compared to that at 0 h control, with error bars representing the standard deviation (SD). *** *p* < 0.001.

As shown in Figure 8, the relative LDH activity (%) at 12 h post-treatment was significantly lower than that at the 0 h control (*p* < 0.001). No significant differences were observed at the other time points (2 h, 4 h, and 6 h) compared to the 0 h control (*p* > 0.05).

## 4. Discussion

Based on the unique morphological appearance, motility patterns, and physiological characteristics of *T. callipaeda*, the TVAS was developed in this study to evaluate its vitality. Moreover, a *T. callipaeda* infection animal model was established, providing a scientific foundation for subsequent research.

Levamisole is an old yet effective anthelmintic that selectively activates nematode acetylcholine-gated ion channels, causing spastic paralysis and subsequent parasite expulsion [27]. In comparison to systemic macrocyclic lactones such as ivermectin, which are often used off label and carry a risk of systemic side effects, for the treatment of dogs, cats, cattle, and sheep, levamisole is used at concentrations between 0.25% and 5% [18,19,20]. A 0.5% levamisole formulation administered in dogs may lead to ocular discomfort [28]. Thus, to maintain efficacy while minimizing potential toxicity, a 5 mg/mL (0.5%) levamisole formulation was chosen for the in vivo antiparasitic experiment. Given the potential irritation to the eye, the maximum number of doses was limited to four. The results showed that administering 5 mg/mL of levamisole every 30 min for four doses effectively killed *T. callipaeda* in the infected rabbit eyes and improved eye symptoms. A single dose of 5 mg/mL levamisole failed to eliminate *T. callipaeda* in the rabbit eyes and instead exacerbated eye symptoms, including increased ocular discharge. Based on the observed phenomena, we hypothesize that the increased ocular discharge in the low-dose group may be due to the expulsion of larvae from the uterus of female *T. callipaeda* under continuous drug stimulation, which could exacerbate ocular inflammation. However, this remains speculative, as no histopathological or inflammatory marker data were collected to support this mechanism. Further investigation is warranted to validate this hypothesis. This result indirectly suggests that a lower concentration or fewer doses may increase ocular discharge in the rabbits, likely due to the expulsion of a large number of larvae from female *T. callipaeda* under drug stimulation rather than due to the drug’s direct irritant effect. As the metabolic mechanism and rate of levamisole in the animal eye are unclear, the in vitro killing results did not fully correspond to the in vivo results, suggesting the need for further investigation into the pharmacokinetic properties of levamisole in the animal eye.

This study scored the ocular symptoms of the experimental rabbits before and after drug administration and also performed blood routine and biochemical tests. Blood tests showed that 5 mg/mL of levamisole administered locally every 30 min for four doses did not cause significant changes or abnormalities in the blood routine or biochemical indices of the experimental rabbits. However, oral or injectable levamisole can cause adverse reactions such as gastrointestinal discomfort, liver and kidney damage, neurological disorders, rashes, and vasculopathy [29,30]. Due to the existence of the ocular–blood barrier, as an ophthalmic solution, it has a short duration of action and a rapid decline in concentration. No notable physiological abnormalities or ocular adverse effects were detected in the experimental rabbits. It can be concluded that this local eye-drop regimen does not have significant systemic effects on the animal’s body.

The sample size used in this study (three rabbits per group) was relatively small, which may reduce the statistical power of our findings. However, due to ethical and logistical constraints, we were limited in the number of animals available for experimentation. Despite this limitation, the results were supported by repeated experiments, and statistical methods were applied to control for variability. Future studies with larger sample sizes are needed to confirm these findings and enhance statistical robustness.

In this study, we evaluated the effects of 5 mg/mL of levamisole on the proliferation and viability of RCECs. The CCK-8 assay results indicated a significant decrease in cell proliferation at 6 h (*p* < 0.0001) and 12 h (*p* < 0.0001) when compared to the baseline (0 h), suggesting that levamisole treatment might inhibit RCEC proliferation over time. However, there was no significant change in cell proliferation at 2 h or 4 h, indicating that the effect of levamisole on cell growth becomes more evident only after prolonged exposure (6 h and 12 h).

The significant reduction in cell proliferation at 6 h and 12 h could be attributed to the cumulative effects of levamisole on cellular metabolism or signaling pathways, which may result in delayed suppression of cell division. These findings suggest that while short-term exposure to levamisole (up to 4 h) does not affect cell proliferation significantly, prolonged exposure leads to a notable decrease in RCEC proliferation. This observation might indicate the onset of a cytostatic effect, where the cells’ ability to divide is inhibited without directly causing cell death.

Although the CCK-8 assay demonstrated a reduction in cell proliferation over time and the LDH assay did not show a corresponding increase in LDH release—suggesting that levamisole does not induce overt cytotoxicity but may modulate cellular activity or proliferation—neither method is inherently able to distinguish between apoptosis, necrosis, or other programmed cell death pathways, and neither can detect early stress responses or sublethal damage. This represents a limitation of the current study. In future work, we plan to incorporate more specific techniques such as live/dead staining, flow cytometry, and TUNEL assays to comprehensively elucidate the cytotoxic mechanisms of levamisole on neuronal and corneal epithelial cells. Levamisole may serve as a more localized, cost-effective topical alternative for ocular thelaziasis. Although our short-term observations revealed no overt toxicity, longer-term topical use on the cornea may pose risks such as epithelial irritation or subclinical inflammation, which merit further evaluation in chronic-use models. To further strengthen the understanding of levamisole’s effects on the eye, future studies should investigate its impact on other relevant ocular cell types, such as corneal or retinal cells, under conditions that more closely mimic the in vivo pharmacokinetics. While the LDH assay results did not show significant membrane damage, the notable decrease in RCEC viability at 6 and 12 h observed in the CCK-8 assay suggests that levamisole may exert cytostatic or delayed cytotoxic effects upon prolonged exposure. These effects might reflect early-stage apoptosis or suppressed cellular metabolism, which are not captured by LDH release alone. Particularly, the reduction in viability at 12 h could be clinically relevant in the context of repeated or long-term topical use. Therefore, although short-term exposure appears safe, caution is warranted for extended applications, and future studies are needed to investigate cumulative toxicity and underlying mechanisms.

In summary, the results suggest that 5 mg/mL of levamisole affects RCEC proliferation in a time-dependent manner, with significant inhibitory effects observed after 6 h and 12 h of treatment. However, the absence of significant cytotoxicity (as indicated by the LDH assay) supports the hypothesis that levamisole may inhibit cell proliferation without causing direct cell death. Future studies are needed to explore the molecular mechanisms underlying this observed effect, particularly the role of levamisole in regulating cellular metabolism and proliferation signaling pathways.

## 5. Conclusions

Topical administration of 5 mg/mL levamisole every 30 min for four doses is effective in treating *T. callipaeda* infection and does not cause significant changes in hematological parameters, biochemical indices, or ocular condition scores in experimental animals. Moreover, 5 mg/mL of levamisole does not significantly affect RCEC proliferation or cellular metabolism within 4 h. However, prolonged exposure (6 h and 12 h) leads to a significant reduction in cell viability and intracellular LDH levels, indicating potential cytotoxic effects over time. These findings suggest that short-term application may be safe, but long-term use requires further evaluation. Future research should focus on chronic-use safety, dose refinement, and the feasibility of translating these results into clinical applications for ocular thelaziasis. There may be broader relevance to ocular parasitic disease control in resource-limited settings, where levamisole’s low cost and wide availability could offer a practical treatment option.

## Figures and Tables

**Table 1:** (**1-1**) Scoring method of TVAS. (**1-2**) Scoring value standards of TVAS.

(**1-1**)			
**Score Items**	**Time**		**Scoring Method**
Light stimulation score	0 s		*T. callipaeda* specimens were removed from a dark environment, images of the morphology immediately were immediately captured after exposure to light and after 30 s of exposure, and then scoring was performed based on the scoring criteria in Table 1(1-2).
	30 s		
Locomotion score	0–30 s		The *T. callipaeda* specimens were transferred from a dark environment, and their locomotion within 30 s of light exposure was recorded via video. The scoring process was conducted following the criteria outlined in Table 1(1-2).
(**1-2**)			
**Score Item**	**Score Value**	**Score Standard**	**Reference Diagram**
Light stimulation score	4	The worm is tightly coiled in multiple loops or irregularly bent with over three bends, each less than 90°.	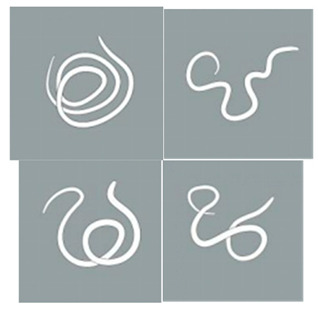
	3	The worm is partially coiled into a single loop, forming shapes that resemble the letters “W”, “E”, or “P” or appears serpentine with 2–3 bends, each less than 90°	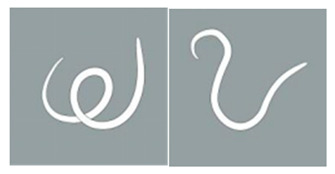
	2	The worm exhibits partial coiling without forming a full circle or presents a single bend with an angle ≤ 160°.	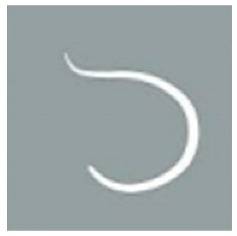
	1	The worm exhibits slight bending or remains straight and rigid, with a curvature exceeding 160°.	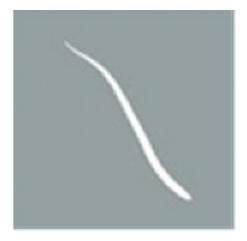
Locomotion score	4	The worm demonstrates more than 5 movements, including serpentine swimming, rolling, and coiling.	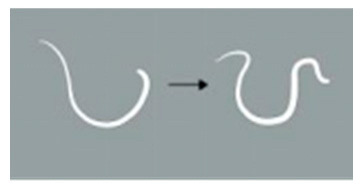
	3	The worm exhibits 3 to 5 movements, including serpentine swimming, rolling, and coiling.	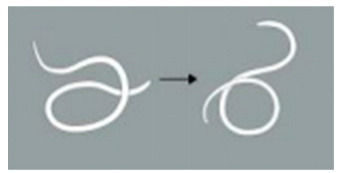
	2	The worm exhibits 1 to 3 movements, including serpentine swimming, rolling, and coiling.	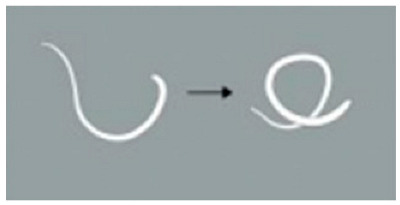
	1	The worm remains static.	/

Note: The schematic pictures in the scoring criteria were hand-painted by Pang Mo, a doctor from China Agricultural University Veterinary Teaching Hospital. The arrows in the figure indicate that *T. callipaeda* moves from the left to the right.

**Table 2:** Scoring standard for ocular symptoms.

Score Item	Score Value	Score Standard
Conjunctiva	0	Conjunctiva appears normal in color with vascularization and no signs of edema.
	1	Conjunctiva is red with vascular congestion and mild edema.
	2	Conjunctiva appears dark red with edema leading to mild eyelid ectropion.
	3	Conjunctiva appears dark red with pronounced edema leading to partial eyelid closure.
	4	Conjunctiva is dark red with indistinct blood vessels and severe edema leading to almost complete eyelid closure.
Discharge	0	No ocular discharge present.
	1	Minimal ocular discharge present.
	2	Noticeable ocular discharge with moisture on the periocular area and eyelashes.
	3	Profuse discharge causing eyelid adhesion.

Note: The conjunctiva encompasses both the palpebral and bulbar conjunctiva. The ocular symptom scoring criteria were developed by the authors, drawing upon commonly used clinical ophthalmic assessment methods [26].

**Table 3:** Average ocular reaction scores of experimental rabbits in different groups at various time points in the in vivo study.

Number of Applications	Score Item	Score Value					
		Control	1 h	2 h	4 h	8 h	12 h
0	Conjunctiva	0	0	0	0	0.7 ± 0.6 *	1 **
	Discharge	0	0	0	0.3 ± 0.6	0.3 ± 0.6	0.3 ± 0.6
1	Conjunctiva	0	0	0	0	1.3 ± 0.6 ***	1 **
	Discharge	0	0	0	0	1.3 ± 0.6 **	1.3 ± 0.6 **
2	Conjunctiva	0	0	0	0	0.7 ± 0.6 *	1 **
	Discharge	0	0	0	0	0.3 ± 0.6	0.3 ± 0.6
3	Conjunctiva	0	0	0	0	0	0.7 ± 0.6 *
	Discharge	0	0	0	0	0.3 ± 0.6	0.7 ± 0.6
4	Conjunctiva	0	0	0	0	0	0
	Discharge	0	0	0	0	0	0

Note: The ocular reaction scores of animals in each treatment group were recorded and statistically compared to those in the control group. Data are expressed as the mean ± SD. Statistical analysis was performed using one-way ANOVA followed by Tukey’s post hoc test. * *p* < 0.05, ** *p* < 0.01, *** *p* < 0.001.

**Table 4:** Hematological results of experimental rabbits after 5 mg/mL of levamisole topical application every 30 min for 4 doses.

Item	Unit	After 24 h of Administration		Before Administration		*t*-Test
		*n*	$\bar{x}$ ± SD	*n*	$\bar{x}$ ± SD	*p*-Value
WBC	10^9^ count·L^−1^	6	7.82 ± 2.13	6	7.86 ± 1.23	*p* = 0.34
NEU	%	6	45.92 ± 11.32	6	45.52 ± 11.12	*p* = 0.62
LYM	%	6	7.82 ± 10.13	6	7.42 ± 9.21	*p* = 0.15
MONO	%	6	2.62 ± 1.17	6	2.42 ± 1.02	*p* = 0.24
EOS	%	6	0.12 ± 0.05	6	0.12 ± 0.10	*p* = 0.52
BASO	%	6	1.12 ± 0.65	6	1.14 ± 0.45	*p* = 0.87
RBC	10^12^ count·L^−1^	6	5.48 ± 0.95	6	5.49 ± 0.85	*p* = 0.12
HGB	g·L^−1^	6	109.38 ± 11.05	6	109.28 ± 10.01	*p* = 0.86
HCT	%	6	33.32 ± 5.03	6	33.22 ± 4.09	*p* = 0.27
MCV	fL	6	62.49 ± 1.52	6	63.49 ± 1.63	*p* = 0.53
MCH	pg	6	21.03 ± 1.10	6	20.13 ± 1.09	*p* = 0.58
MCHC	g·L^−1^	6	314.21 ± 10.65	6	314.23 ± 10.21	*p* = 0.36
RDW	%	6	12.42 ± 0.41	6	12.35 ± 0.19	*p* = 0.50
PLT	10^9^ count·L^−1^	6	142.41 ± 24.35	6	146.31 ± 22.15	*p* = 0.89
PCT	%	6	0.19 ± 0.05	6	0.21 ± 0.06	*p* = 0.12
MPV	fL	6	5.02 ± 0.92	6	4.98 ± 0.22	*p* = 0.37
PDW	fL	6	15.33 ± 1.02	6	15.23 ± 1.12	*p* = 0.62

n represents the number of experimental rabbits.

**Table 5:** Blood biochemistry results of rabbits after topical administration of 5 mg/mL levamisole (4 times at 30 min intervals).

Item	Unit	After 24 h of Administration		Before Administration		*t*-Test
		*n*	$\bar{x}$ ± SD	*n*	$\bar{x}$ ± SD	*p*-Value
TP	g·L^−1^	6	66.22 ± 4.03	6	65.23 ± 4.10	*p* = 0.46
ALB	g·L^−1^	6	42.32 ± 4.22	6	43.52 ± 3.12	*p* = 0.67
GLB	g·L^−1^	6	27.32 ± 3.23	6	26.89 ± 0.43	*p* = 0.74
A/G	%	6	1.63 ± 0.13	6	1.70 ± 0.09	*p* = 0.45
ALT	U·L^−1^	6	1.42 ± 0.25	6	1.51 ± 0.03	*p* = 0.67
AST	U·L^−1^	6	45.32 ± 15.25	6	45.40 ± 13.85	*p* = 0.23
ALP	U·L^−1^	6	96.28 ± 31.25	6	96.25 ± 30.13	*p* = 0.47
CK	U·L^−1^	6	1049.32 ± 431.25	6	1049.59 ± 420.32	*p* = 0.99
GLU	mmol L^−1^	6	7.32 ± 0.53	6	7.31 ± 0.42	*p* = 0.36
BUN	mmol L^−1^	6	8.39 ± 1.32	6	8.28 ± 1.25	*p* = 0.38
CRE	mmol L^−1^	6	111.32 ± 21.50	6	113.42 ± 22.01	*p* = 0.43
Ca	mmol L^−1^	6	3.31 ± 0.75	6	3.36 ± 0.43	*p* = 0.24
*p*	mmol L^−1^	6	1.52 ± 0.31	6	1.57 ± 0.03	*p* = 0.66
CHOL	mmol L^−1^	6	1.39 ± 0.45	6	1.43 ± 0.39	*p* = 0.57
TG	mmol L^−1^	6	0.89 ± 0.45	6	0.95 ± 0.02	*p* = 0.93

## Data Availability

The original contributions presented in this study are included in the article. Further inquiries can be directed to the corresponding authors.

## Abbreviations

*T. callipaeda*	*Thelazia callipaeda*
RCEC	rabbit conjunctival epithelial cells
TVAS	*Thelazia callipaeda* viability assessment system
NS	normal saline
DW	distilled water
WBC	white blood cell count
NEU	neutrophil
LYM	lymphocyte
MONO	monocyte
EOS	eosinophil
BASO	basophil
RBC	red blood cell count
HGB	hemoglobin
HCT	hematocrit
MCV	mean corpuscular volume
MCH	mean corpuscular hemoglobin concentration
MCHC	mean corpuscular hemoglobin
RDW	red cell distribution width
PLT	platelet
PCT	thrombocytocrit
MPV	mean platelet volume
PDW	platelet distribution width
TP	total protein
ALB	albumin
GLB	globulin
A/G	albumin/globulin
ALT	alanine aminotransferase
AST	aspartate aminotransferase
ALP	alkaline phosphatase
CK	creatine kinase
GLU	glucose
BUN	blood urea nitrogen
CRE	creatinine
CHOL	cholesterol
TG	triglyceride
OD	optical density
